# Psychiatric Presentation of Anti-NMDA Receptor Encephalitis

**DOI:** 10.3389/fneur.2019.01086

**Published:** 2019-11-05

**Authors:** Dominique Endres, Sebastian Rauer, Winfried Kern, Nils Venhoff, Simon J. Maier, Kimon Runge, Patrick Süß, Bernd Feige, Kathrin Nickel, Timo Heidt, Katharina Domschke, Karl Egger, Harald Prüss, Philipp T. Meyer, Ludger Tebartz van Elst

**Affiliations:** ^1^Section for Experimental Neuropsychiatry, Department of Psychiatry and Psychotherapy, Medical Center - University of Freiburg, Faculty of Medicine, University of Freiburg, Freiburg, Germany; ^2^Department of Psychiatry and Psychotherapy, Medical Center - University of Freiburg, Faculty of Medicine, University of Freiburg, Freiburg, Germany; ^3^Department of Neurology, Medical Center - University of Freiburg, Faculty of Medicine, University of Freiburg, Freiburg, Germany; ^4^Division of Infectious Diseases, Department of Medicine II, Medical Center - University of Freiburg, Faculty of Medicine, University of Freiburg, Freiburg, Germany; ^5^Department of Rheumatology and Clinical Immunology, Medical Center - University of Freiburg, Faculty of Medicine, University of Freiburg, Freiburg, Germany; ^6^Department of Molecular Neurology, University Hospital Erlangen, Erlangen, Germany; ^7^University Heart Center Freiburg, Department of Cardiology and Angiology I, Medical Center - University of Freiburg, Faculty of Medicine, University of Freiburg, Freiburg, Germany; ^8^Centre for Basics in Neuromodulation, Faculty of Medicine, University of Freiburg, Freiburg, Germany; ^9^Department of Neuroradiology, Medical Center - University of Freiburg, Faculty of Medicine, University of Freiburg, Freiburg, Germany; ^10^Department of Neurology and Experimental Neurology, Charité - Universitätsmedizin Berlin, Berlin, Germany; ^11^German Center for Neurodegenerative Diseases (DZNE) Berlin, Berlin, Germany; ^12^Department of Nuclear Medicine, Medical Center - University of Freiburg, Faculty of Medicine, University of Freiburg, Freiburg, Germany

**Keywords:** anti-NMDA receptor encephalitis, encephalopathy, autoimmune psychosis, antibodies, steroids, vaccination

## Abstract

**Background:** Anti-N-methyl D-aspartate (NMDA) receptor encephalitis is an autoimmune condition characterized by neuropsychiatric symptoms, including epileptic seizures, movement disorders, autonomic instability, disturbances of consciousness, paranoia, delusions, and catatonia. Ovarian teratomas and viral infections, typically Herpes simplex viruses, have previously been demonstrated to precipitate anti-NMDA receptor encephalitis, but in many cases, the trigger remains unclear. The detection of anti-NMDA receptor antibodies in cerebrospinal fluid (CSF), in combination with other CSF, electroencephalography (EEG), or magnetic resonance imaging (MRI) abnormalities, typically leads to diagnostic clarification.

**Case Presentation:** We present the case of a 22-year-old female patient who developed an acute polymorphic psychotic episode 3 days after receiving a booster vaccination against tetanus, diphtheria, pertussis, and polio (Tdap-IPV). Her psychiatric symptoms were initially diagnosed as a primary psychiatric disorder. Her MRI, EEG, and CSF results were non-specific. Anti-NMDA receptor IgG antibodies against the GluN1 subunit were detected in her serum (with a maximum titer of 1:320), but not in her CSF. [18F]fluorodeoxyglucose positron emission tomography (FDG-PET) showed pronounced relative hypermetabolism of her association cortices and a relative hypometabolism of the primary cortices, on the basis of which an anti-NMDA receptor encephalitis diagnosis was made, and treatment with a steroid pulse was initiated. The treatment led to fast and convincing clinical improvement with normalization of neuropsychological findings, considerable improvement of FDG-PET findings, and decreasing antibody titers.

**Conclusion:** The patient's psychiatric symptoms were most likely caused by anti-NMDA receptor encephalitis. Her polymorphic psychotic symptoms first occurred after she had received a Tdap-IPV booster vaccination. Although the vaccination cannot have caused the initial antibody formation since IgG serum antibodies were detected only 3 days after administration of the vaccine, the vaccine may have exerted immunomodulatory effects. MRI, EEG, and CSF findings were non-specific; however, FDG-PET identified brain involvement consistent with anti-NMDA receptor encephalitis. This case shows the importance of implementing a multimodal diagnostic work-up in similar situations. The negative CSF antibody finding furthermore fits to the hypothesis that the brain may act as an immunoprecipitator for anti-NMDA receptor antibodies.

## Background

Anti-N-methyl D-aspartate (NMDA) receptor encephalitis was first identified as a paraneoplastic disorder associated with ovarian teratoma (1). Since then, large case series with hundreds of cases have allowed researchers to describe its typical clinical course (2). After a prodromal phase with flu-like symptoms, behavioral abnormalities and acute polymorphic symptoms, often accompanied by catatonic symptoms, are typically observed. In addition, movement disorders, such as dyskinesia or rigidity, autonomous instability, or central hypoventilation, typically occur as the disease progresses, as do disturbances of consciousness. Epileptic seizures may also occur at any time (3, 4). Thus, the typical clinical syndrome is neuropsychiatric in nature and thereby points to an organic cause, but there are occasional cases that present like primary idiopathic psychiatric disorders, typically with catatonia (5, 6). Young women are most frequently affected (2).

Patients with anti-NMDA receptor encephalitis usually have IgG antibodies against the GluN1 subunit of NMDA receptors in their cerebrospinal fluid (CSF) (7–10). Electroencephalography (EEG) results are usually abnormal (3), and in some patients a specific EEG phenomenon, the so-called “extreme delta brush,” has been observed (11). It is well-known that ovarian teratomas and viral infections can trigger autoimmune reactions. In paraneoplastic cases associated with ovarian teratomas, the tumor itself contains NMDA receptors, which are typically found in brain tissue, and this seems to trigger the encephalitic autoimmune response (3). In addition, most authors suggest that virus-induced neuroinflammation can trigger antibody formation. While Herpes simplex is most widely blamed in this context, other viruses or bacteria (e.g., varicella zoster, mycoplasma) may also potentially trigger the encephalitic autoimmune response (3). However, often no specific causes for antibody production can be identified.

Vaccinations may also play a role as unspecific triggers (12, 13). A PubMed search for “vaccination and NMDA” (on 24 April 2019) identified 27 published articles addressing this issue, including two case reports. The first case was reported to have developed anti-NMDA receptor encephalitis 5 weeks after a booster vaccination against tetanus, diphtheria, pertussis, and polio (Tdap-IPV) (14). In the second, a patient was reported as having developed a postural tachycardia syndrome with anti-NMDA receptor antibodies after receiving a human papillomavirus vaccination (15). An additional vaccine-related case was also mentioned in a publication by Wang (12), who reported that an individual developed anti-NMDA receptor encephalitis after receiving a vaccination against Japanese encephalitis. A further search for “vaccination and autoimmune encephalitis” resulted in only 12 results and no case reports.

## Case Presentation

Here, we present the case of a 22-year-old female German patient who experienced an acute polymorph psychotic episode after receiving a Tdap-IPV booster vaccination (Repevax®). The patient received the vaccination at the beginning of January 2019 and reported an immediate “strange” feeling. She rapidly developed flu-like symptoms (headache, jittering), and 3 days later, she was admitted to a hospital emergency department with psychosis-like symptoms: She was hearing her thoughts and developed paranoia (e.g., she felt that she was being used as a test subject by the clinic staff). Her neurological and general medical examinations were normal, and her CSF samples, magnetic resonance imaging (MRI) scans of her brain, and the EEG showed no relevant abnormalities. Anti-NMDA receptor IgG antibodies against the GluN1 subunit (titer 1:80 3 days after symptom-onset; titer 1:320 after 6 days, both times using biochip-assays) were detected in her serum, but the CSF antibody testing was negative (3 days after symptom-onset, not performed after 6 days; using cell-based assay). After 6 days, the patient was discharged from the hospital and began outpatient psychotherapy with the assumption that her problems were caused by being separated from her parental home. In the meantime, the patient stopped her vocational training and moved house back to her parents. The mother reported that her daughter behaved like a toddler and that she was helpless, unbalanced, and dependent. She was readmitted to the hospital 47 days after vaccination due to her persistent symptomatology with strong anxiety and ambivalence. Treatment with lorazepam (2 mg) led to a reduction in her anxiety.

Fifty-three days after the vaccination, the patient presented herself to our hospital. At this time, she reported that she had pronounced anxiety, an inability to make decisions, strong restlessness, and sensory overload, and that she was emotionally unstable (i.e., she could suddenly burst into tears) and had cognitive deficits (i.e., was not able to keep conversations for a longer period of time in a concentrated manner). She also reported difficulties in falling asleep and sleeping through the night, reduced appetite, and a weight loss of 4–5 kg over 2 months. In addition, she reported experiencing burning sensations on her chest and head and intermittent tachycardia.

### Diagnostic Findings

**Figure 1:**
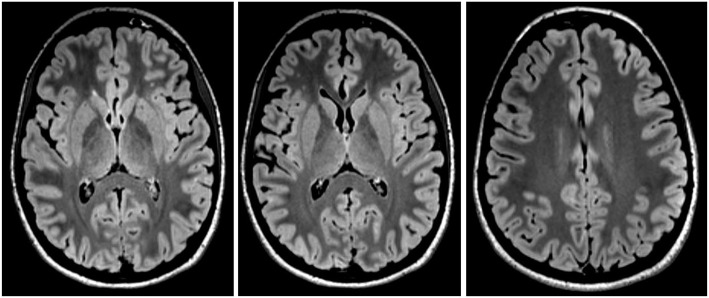
Magnetic resonance imaging showed slight, non-specific bifrontal medullary lesions, but was otherwise inconspicuous.

**Figure 2:**
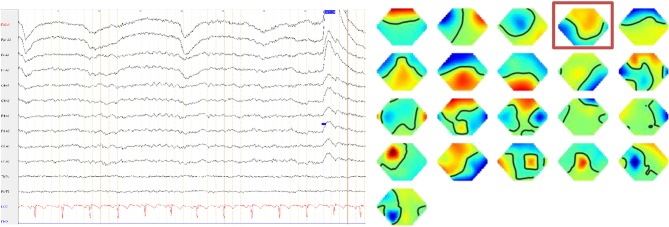
The electroencephalography depicted a ß-rhythm, with an intermittently slow wave activity of 6–7/s. The independent component analyses showed a frontocentral topography (component 4, framed) compatible with tiredness.

**Figure 3:**
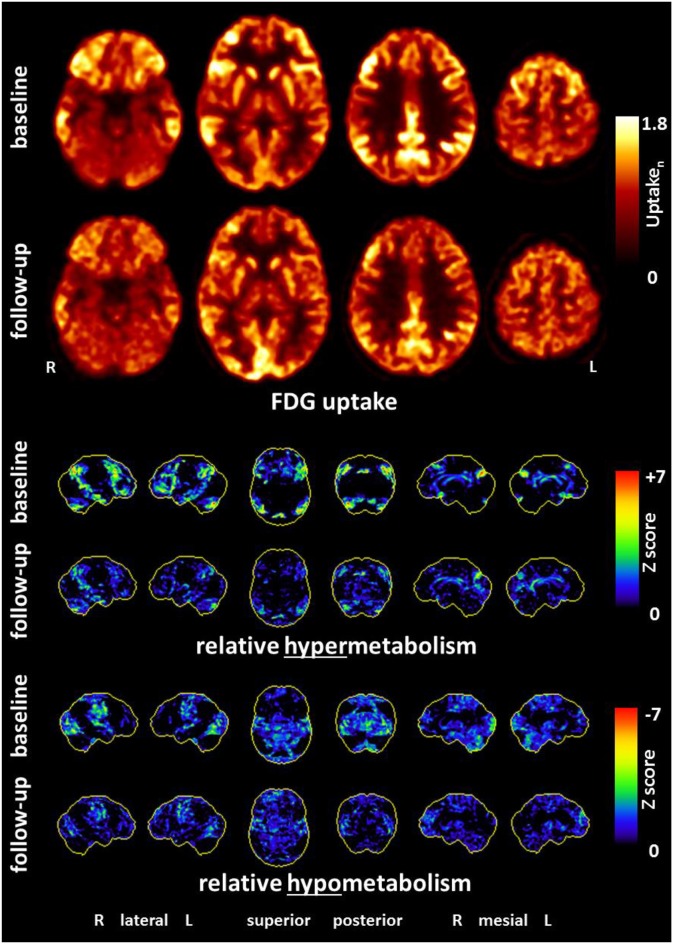
An [18F]fluorodeoxyglucose positron emission tomography (FDG-PET) examination at baseline showed a pronounced relative hypermetabolism of parts of the association cortices and mild relative hypometabolism of the primary cortices, consistent with encephalitis. These metabolic findings markedly improved at follow-up. **(Upper panel)** Transaxial FDG-PET images at baseline and follow-up. **(Middle and lower panel)** Metabolic deviations from healthy controls, given as three-dimensional stereotactic surface projections (3D-SSP), color-coded as Z score (middle, relative metabolic increases; lower, relative metabolic decreases). All analyses were performed with Neurostat/3D-SSP [LIT] (16).

**Figure 4:**
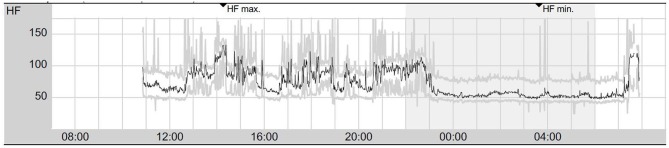
Twenty-four hours of electrocardiogram showing signs of vegetative dysbalance with intermittent sinus tachycardia during daytime and sinus rhythm with stable frequencies during the night (presented are mean heart rates per minute). HF, heart rate; HFmax, maximum heart rate; HFmin, minimum heart rate.

**Figure 5:**
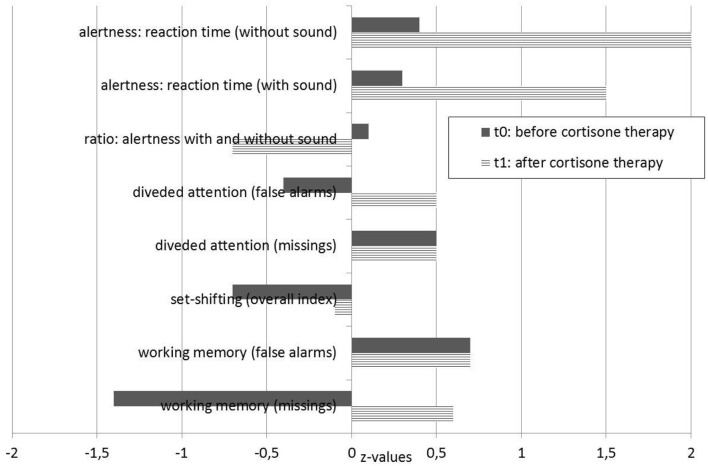
Neuropsychological test results. t0 testing was performed before treatment, and t1 testing was performed 14 days after the steroid pulse treatment.

A high-resolution 3 Tesla structural MRI of her brain was normal except for a few slight, non-specific bifrontal white matter lesions (Figure 1). An EEG depicted a β-rhythm (likely related to lorazepam intake), with rare intermittent slow wave activity of 6–7/s. Independent Component Analysis (ICA) showed a frontocentral topography compatible with tiredness (Figure 2). Her CSF basic analysis results were inconspicuous (normal white blood cell count/albumin quotient/protein concentration, no CSF specific oligoclonal bands). In her serum, anti-NMDA receptor GluN1-IgG antibodies were still positive (titer 1:160, using cell-based-assays), while IgA and IgM antibodies were negative. CSF antibody testing was again negative, including biochip assays, cell-based assays, and tissue testing. An [18F]fluorodeoxyglucose positron emission tomography (FDG-PET) examination showed a pronounced relative hypermetabolism of parts of the association cortices and a relative hypometabolism of the primary cortices, well compatible with encephalitis (Figure 3). A whole-body FDG-PET/CT detected no lesions suspicious of malignancy, including no ovarian teratoma. A gynecologic ultrasound and an MRI of the abdomen were unremarkable. A long-term electrocardiogram (ECG) showed recurrent sinus tachycardia that stopped at night (Figure 4). Neuropsychological testing showed significant deficits in working memory (Figure 5). All diagnostic findings are summarized in Table 1.

**Table 1:** Diagnostic findings (~8 weeks after onset of symptoms).

Physical examination	Inconspicuous neurological and general medical examinations.
Basic blood/urine analyses	Blood cell count, electrolytes, liver/kidney/pancreas values, vitamin B12, and selenium were normal. Vitamin D was normal, but not optimal (28.9 ng/ml; optimal: >30 ng/ml), folic acid was reduced (4.1 ng/ml; reference ≥4.8 ng/ml). Thyroid-stimulating hormone, triiodothyronine, and thyroxine levels were in normal ranges. The urine analyses showed normal findings, screening on illegal drugs was negative.
Immunological blood testing	No antibodies against the intracellular onconeural antigens Yo, Hu, CV2/CRMP5, Ri, Ma1/2, SOX1, or the intracellular synaptic antigens GAD65/amphiphysin were found (using Ravo line assay). Antibodies against different neuronal cell surface antigens (*AMPA-R, GABA-B-R, VGKC-complex [LGI1, Caspr2]*) were negative (using biochip-assays). IgG anti-NMDA-R antibodies were positive (titer 1:160 using cell-based assay [reference <1:20] and 1:32 [++, reference <1:10] using biochip-assay), anti IgA and IgM antibodies were negative (using biochip-assays). Negative tissue testing for known and unknown antineuronal antibodies. Aquaporin 4 and MOG antibodies were negative. Autoantibodies against thyroglobulin, TSH receptor, and thyroid peroxidase were not increased. Screening for antinuclear antibodies (ANA) in IIF was normal. Anti-neutrophil cytoplasmic antibodies, antiphospholipid antibodies, rheumatoid factor, and anti-mitochondrial antibodies were negative. CH50 was slightly increased (119, reference: 65–115%), no other changes in the complement system (C3, C4, CH50, C3d) were observed. Normal serum IgA, IgM, and IgG immunoglobulin concentrations; immunofixation showed no monoclonal antibody production. B-cell panel showed no relevant pathologies beside of slightly reduced percentage of lymphocytes (24.1%, reference: 27–34%). All other cell counts were normal.
Infectiological blood testing	Serologies for Lyme borreliosis, syphilis, and HIV were negative. The vaccination titers showed the following values: Anti-tetanus toxoid IgG: >5 IU/ml (positive from >0.1 IU/ml), diphtheria -antitoxin IgG: >2 IU/ml (positive from >0.1 IU/ml), anti-pertussis IgG: 154.67 IU/ml (positive from >50 IU/ml), anti-polio 1: >1:512 (sufficient protection), and anti-polio 3: >1:512 (sufficient protection).
Cerebrospinal fluid analyses	Normal white blood cell count (1/μL; reference <5/μL). Normal protein concentration (207 mg/L; reference <450 mg/L), and normal age-corrected albumin quotient: 2.4; age-dependent reference <6.5 ×10^−3^). No CSF specific oligoclonal bands; IgG index not increased (0.49; reference ≤ 0.7). CSF lactate not increased (1.39 mmol/l; reference 1, 5–2, 1 mmol/L). Antibodies against neuronal cell surface antigens (*NMDAR, AMPA-R, GABA-B-R, VGKC-complex [LGI1, Caspr2]*) were negative (Biochip assay). The live-cell assay and the tissue antibody test was also negative.
Cerebral magnetic resonance imaging	Inconspicuous, except for a few slight, non-specific bifrontal medullary lesions.
Electroencephalography	β-rhythm (appropriate due to lorazepam intake), with an intermittently slow wave activity of 6–7/s. The independent component analyses showed a frontocentral topography (component 4, framed) compatible with tiredness.
[^18^F]fluorodeoxyglucose positron emission tomography	Pronounced relative hypermetabolism of parts of the association cortices and a relative hypometabolism of the primary cortices. No lesion suspicious of malignancy on whole-body PET/CT.
Ophthalmological examinations	Ophthalmological examinations were inconspicuous. Macular scans performed using SPECTRALIS^®^ optical coherence tomography device (spectral-domain OCT) showed normal retinal volume and normal full retina thickness in all subfields with reference to normative data given by Nieves-Moreno et al. (17).
Heart examinations	Inconspicuous resting electrocardiography (ECG). The long-term ECG measurement showed a sinus rhythm with intermittent sinus tachycardic pulse; no relevant pauses or severe cardiac arrhythmia.

### Illness, Somatic, and Family Histories

The patient's history was negative for *in-utero* or birth complications, febrile convulsions, craniocerebral traumata, or inflammatory brain diseases. During her first two decades of life, she had no evidence of a developmental disorder such as autism spectrum syndrome, attention deficit hyperactivity disorder, or a tic syndrome. The patient described herself as a rather anxious and insecure person; however, she clearly did not fulfill the criteria for any personality, affective, or anxiety disorder. She had no history of cancer or of autoimmune, neurological, or other somatic disorders. Apart from her paternal grandfather having suffered from Alzheimer's disease she had no family history of psychiatric disorders (including her siblings, parents, and grandparents). Her maternal grandmother had died of a pancreatic carcinoma, her maternal grandfather had died of an unspecified tumor at the age of 38.

### Treatment and Outcome

Lorazepam led to a slight improvement in the patient's anxiety symptoms. After pausing the medication at the patient's request, anxiety symptoms became more severe. We then treated her with 7.5 mg diazepam. A steroid pulse (5 ×500 mg methylprednisolone, with oral tapering from 40 mg and halving every week over 4 weeks; further reductions took place in 2 mg steps) was performed for immunological treatment. Directly after the steroid pulse, we were able to discontinue diazepam without any worsening of the patient's anxiety symptoms. On the 5th day of the steroid pulse, the patient reported that she felt well again. Over the following 2 weeks, her fears and ambivalence were greatly reduced, her emotions stabilized, and neither inner restlessness nor stimulus overload occurred. A slight form of sleeping disorder persisted; however, her appetite improved. The burning sensations in the chest and head no longer occurred. Follow-up cognitive testing results were within the normal range (Figure 5), and a follow-up FDG-PET (20 days after starting the steroid pulse) showed considerable improvements of the metabolic findings (Figure 3). Her anti-NMDA IgG antibody titers decreased (1:40 ~1 week after steroid pulse and 1:80 ~2 weeks after steroid pulse treatment, using cell-based assays in both analyses). Approximately half a year after the steroid pulse treatment, the patient was completely free of symptoms.

## Discussion

Our case report describes a female patient with anti-NMDA receptor encephalitis with isolated psychiatric manifestation presenting with acute polymorphic psychotic symptoms that developed directly after the patient had received a Tdap-IPV booster vaccination, most probably in the context of preexisting anti-NMDA receptor IgG antibodies.

### Diagnostic and Pathophysiological Considerations

Basic diagnostic procedures using EEG, MRI, and CSF examinations only showed non-specific findings. While the patient initially displayed acute polymorphic psychotic symptoms, she did not present the typical neurological symptoms of encephalitis or encephalopathy, such as movement disorders or epileptic seizures, and her psychiatric symptoms were initially misdiagnosed as a primary mental disorder. However, her vegetative symptom (sinus tachycardia) was typical for anti-NMDA receptor encephalitis, possibly caused by catecholaminergic hyperstimulation, similar to the well-known ketamine effects (18). However, since the sinus tachycardia only occurred during the day, it was more likely due to the patient's anxiety than to a primarily antibody-induced genesis. There were no indications of cancer, particularly ovarian teratoma, neither from the whole-body FDG-PET/CT nor from the gynecological examination. Also, there was no observed viral association nor any preexisting immunological predisposition.

The combination of GluN1-IgG antibodies in the patient's serum and the FDG-PET finding of relative hypermetabolism in the patient's association cortex and a relative hypometabolism of the primary cortices led us to the probable diagnosis of anti-NMDA receptor encephalitis (19). Primary vaccination-induced encephalitis seemed less likely due to the clinical course and the FDG-PET findings, the latter of which were consistent with anti-NMDA receptor encephalitis (20–22), including an increased frontal to occipital metabolic ratio that markedly improved upon treatment (23). Indeed, the present patient showed strong frontal hypermetabolism and occipital hypometabolism at baseline, which resolved almost entirely after treatment.

However, due to the timing of symptom onset, there was a possible association with the Tdap-IPV booster vaccination. The presence of isolated anti-NMDA receptor IgG antibodies in the acute phase (IgG antibodies are typically not detectable in the first week after antigen contact) and the rapid onset of symptoms after vaccination clearly indicate the presence of preexisting antibodies or latent, non-clinically manifest encephalitis. Therefore, the vaccination cannot be the initial reason for anti-NMDA receptor antibody production. However, vaccinations physiologically lead to an immunological response with T-cell and B-cell activation. Preexisting specific T- and B-lymphocyte clones might have been re-stimulated, causing them to proliferate and leading to excessive antibody synthesis. Indeed, recent work suggested that antibody-secreting cells releasing functional anti-NMDA-R antibodies are part of the human naïve B cell repertoire and might thus be much more common than previously assumed (24). It is possible that the vaccine's effect of boosting antibody production led to a short-term increase in the patient's anti-NMDA receptor antibody levels, which could in turn have caused her clinical symptoms. One could also speculate that a temporary dysfunction of the blood-brain-barrier function might have led to increased antibody levels in the central nervous system (CNS). In summary, it can be hypothesized that too many anti-NMDA-R antibodies were present in the CNS for a short time and that this has led to a reversible internalization of the anti-NMDA receptors, thereby altering glutamatergic signaling and, consequently, changing neuronal glucose metabolism, with the described FDG-PET alterations and psychiatric symptoms [c.f. (6, 8, 25)]. The FDG-PET finding probably does not reflect classic neuroinflammation, so it may be better to speak of encephalopathy rather than encephalitis in such cases [cf. (8, 26)].

Normally, antibody detection in CSF characterizes anti-NMDA receptor encephalitis (7–9). The negative CSF result in the patient presented here is nonetheless valid because neither the biochip nor cell-based assays nor the tissue tests were conspicuous. The inconspicuous CSF finding could be due to the relatively low antibody levels compared to typical anti-NMDA receptor encephalitis with broader neurological symptoms (i.e., epileptic seizures). In the present constellation, it is plausible that all antibodies reaching the CNS compartment were bound to brain tissue. Laboratory experiments have shown that the brain can act as an immunoprecipitator for anti-NMDA receptor antibodies (27). Such cases with negative CSF findings could be associated with milder psychiatric manifestations. A similar situation has been found in neuromyelitis optica spectrum disorders: Aquaporin-4 (AQP4) and myelin oligodendrocyte glycoprotein (MOG) antibodies develop in the periphery, but if AQP4 or MOG antibodies reach the CNS, they can have pathological effects. They also typically cannot be detected in the CSF, and even oligoclonal bands are usually negative in those affected (28).

To date, only one comparable report of an association between anti-NMDA receptor antibodies and Tdap-IPV booster vaccination has been published, in which a 15-year-old boy developed anti-NMDA receptor encephalitis 5 weeks after receiving the vaccination. This previous patient presented with a severe neuropsychiatric syndrome with dyskinesia and choreatiform movements, and antibodies were also detected in his CSF; however, detailed information about antibody type was not reported (14).

### Clinical Implications

A study of the Tdap vaccination without the additional inclusion of IPV found that there were neurological complications in 41 out of 2,090 vaccinated persons, including three who suffered from encephalitis/encephalopathy. The same study also reported demyelinating disorders (*N* = 4), seizures (*N* = 7), Bell's palsy (*N* = 7), and Guillain-Barré syndrome (*N* = 10) (29). Future research should investigate whether patients who report neurological side effects after receiving the Tdap-IPV booster vaccination are positive for anti-NMDA receptor antibodies. Antibody status could then be used to warn patients against vaccination side effects.

In addition, our case shows that an inconspicuous CSF anti-NMDA receptor antibody status does not rule out brain involvement. Despite non-specific MRI, EEG, and CSF findings, profound metabolic changes suggestive of encephalitis may be detected using FDG-PET. This case therefore demonstrates the importance of using multiple modes of examination to clarify and confirm or reject diagnosis in patients who are seropositive for anti-NMDA receptor antibodies and who have acute polymorphic psychotic episodes. The broad spectrum of additional examinations (EEG, MRI, CSF, FDG-PET) were of great relevance for our patient, since a steroid pulse treatment resulted in rapid clinical improvement. We discussed with the patient the possibility of additional temporary maintenance therapy with azathioprine or rituximab, but the patient chose not to receive this treatment due to her clinical improvement. Further research should investigate how patients with similar diagnostic criteria and symptoms can best be treated over longer periods of time.

### Limitations

Although the pathophysiological considerations discussed here are plausible, they cannot be definitively proven by this case report since the possible disease-causing effects of anti-NMDA receptor antibodies could only be shown indirectly via FDG-PET changes.

## Conclusions

This case report shows a possible association between vaccination and the immediate development of anti-NMDA receptor encephalitis in a young female patient with likely preexisting anti-NMDA receptor antibodies. Since IgG antibodies were already initially detectable, the vaccination cannot have led to the initial antibody formation, but it may have exerted immunomodulatory effects. The further particularities of our case are the patient's mild psychiatric manifestation that mimicked an idiopathic psychiatric syndrome, her non-specific basic diagnostics (MRI, EEG, and CSF), and ultimate diagnostic clarification by FDG-PET. Detection of an immunological cause was essential, since it suggested immunosuppressive treatment, which led to rapid clinical improvement.

## Data Availability Statement

All necessary data can be found in the paper.

## Ethics Statement

Written informed consent was obtained for the publication of any images or data included in this article.

## Consent for Publication

The patient has given her signed written informed consent for this case report, including the presented images, to be published.

## Author Contributions

DE performed the data research, and wrote the paper. DE and LT treated the patient. SR performed the neurological interpretation. HP supported the neurological interpretation and performed the tissue antibody tests. BF and LT performed the EEG analyses. WK performed the infectiological interpretation. TH interpreted the cardiological findings. PM was responsible for the nuclear medicine investigations and interpretation. KE and SM performed and interpreted the MRIs. NV performed the rheumatological measurements and interpreted the results. KR, PS, KN, KD, and LT supported the clinical interpretation. All authors were critically involved in the theoretical discussion and composition of the manuscript. All authors read and approved the final version of the manuscript.

### Conflict of Interest

SR: receiving consulting and lecture fees, grant, and research support from Bayer Vital, Biogen, Merck Serono, Novartis, Sanofi-Aventis, Genzyme, Roche, and Teva. Furthermore, SR indicates that he is a founding executive board member of Ravo Diagnostika GmbH Freiburg. LT: advisory boards, lectures, or travel grants within the last 3 years: Eli Lilly, Janssen-Cilag, Novartis, Shire, UCB, GSK, Servier, Janssen, and Cyberonics. The remaining authors declare that the research was conducted in the absence of any commercial or financial relationships that could be construed as a potential conflict of interest.

## References

[1] Dalmau J Tüzün E Wu HY Masjuan J Rossi JE Voloschin A. Paraneoplastic anti-N-methyl-D-aspartate receptor encephalitis associated with ovarian teratoma. Ann Neurol. (2007) 61:25–36. 10.1002/ana.21050 17262855 PMC2430743

[2] Titulaer MJ McCracken L Gabilondo I Armangué T Glaser C Iizuka T. Treatment and prognostic factors for long-term outcome in patients with anti-NMDA receptor encephalitis: an observational cohort study. Lancet Neurol. (2013) 12:157–65. 10.1016/S1474-4422(12)70310-1 23290630 PMC3563251

[3] Dalmau J Geis C Graus F. Autoantibodies to synaptic receptors and neuronal cell surface proteins in autoimmune diseases of the central nervous system. Physiol Rev. (2017) 97:839–87. 10.1152/physrev.00010.2016 28298428 PMC5539405

[4] Gurrera RJ. Recognizing psychiatric presentations of anti-NMDA receptor encephalitis in children and adolescents: a synthesis of published reports. Psychiatry Clin Neurosci. (2019) 73:262–8. 10.1111/pcn.12821 30653785

[5] Kayser MS Titulaer MJ Gresa-Arribas N Dalmau J. Frequency and characteristics of isolated psychiatric episodes in anti–N-methyl-d-aspartate receptor encephalitis. JAMA Neurol. (2013) 70:1133–9. 10.1001/jamaneurol.2013.3216 23877059 PMC3809325

[6] Endres D Perlov E Stich O Rauer S Maier S Waldkircher Z. Hypoglutamatergic state is associated with reduced cerebral glucose metabolism in anti-NMDA receptor encephalitis: a case report. BMC Psychiatry. (2015) 15:186. 10.1186/s12888-015-0552-4 26231521 PMC4522073

[7] Leypoldt F Armangue T Dalmau J. Autoimmune encephalopathies. Ann NY Acad Sci. (2015) 1338:94–114. 10.1111/nyas.12553 25315420 PMC4363225

[8] Endres D Bechter K Prüss H Hasan A Steiner J Leypoldt F. [Autoantibody-associated schizophreniform psychoses: clinical symptomatology]. Nervenarzt. (2019) 90:547–63. 10.1007/s00115-019-0700-z 30968197

[9] Tebartz van Elst L Bechter K Prüss H Hasan A Steiner J Leypoldt F. Autoantibody-associated schizophreniform psychoses: pathophysiology, diagnostics, and treatment. Nervenarzt. (2019). 90:745–61. 10.1007/s00115-019-0735-1 31197396

[10] Tebartz van Elst L Süß P Endres D Autoimmunenzephalitiden in der Psychiatrie. CME-fortbildung. InFo Neurol Psychiatrie. (2019). 10.1007/s15005-019-0022-5

[11] Schmitt SE Pargeon K Frechette ES Hirsch LJ Dalmau J Friedman D. Extreme delta brush: a unique EEG pattern in adults with anti-NMDA receptor encephalitis. Neurology. (2012) 79:1094–100. 10.1212/WNL.0b013e3182698cd8 22933737 PMC3525298

[12] Wang H. Anti-NMDA receptor encephalitis and vaccination. Int J Mol Sci. (2017) 18:E193. 10.3390/ijms18010193 28106787 PMC5297824

[13] Wang H. A protocol for investigating the association of vaccination and anti-NMDA receptor encephalitis. Front Biosci. (2018) 10:229–37. 10.2741/s511 28930529

[14] Hofmann C Baur MO Schroten H Anti-NMDA receptor encephalitis after TdaP-IPV booster vaccination: cause or coincidence? J Neurol. (2011) 258:500–1. 10.1007/s00415-010-5757-3 20878418

[15] Blitshteyn S Brook J. Postural tachycardia syndrome (POTS) with anti-NMDA receptor antibodies after human papillomavirus vaccination. Immunol Res. (2017) 65:282–4. 10.1007/s12026-016-8855-1 27561785

[16] Minoshima S Frey KA Koeppe RA Foster NL Kuhl DE. A diagnostic approach in Alzheimer's disease using three-dimensional stereotactic surface projections of fluorine-18-FDG PET. J Nucl Med. (1995) 36:1238–48. 7790950

[17] Nieves-Moreno M Martínez-de-la-Casa JM Cifuentes-Canorea P Sastre-Ibáñez M Santos-Bueso E Sáenz-Francés F. Normative database for separate inner retinal layers thickness using spectral domain optical coherence tomography in Caucasian population. PLoS ONE. (2017) 12:e0180450. 10.1371/journal.pone.0180450 28678834 PMC5498048

[18] Dalmau J Gleichman AJ Hughes EG Rossi JE Peng X Lai M. Anti-NMDA-receptor encephalitis: case series and analysis of the effects of antibodies. Lancet Neurol. (2008) 7:1091–8. 10.1016/S1474-4422(08)70224-2 18851928 PMC2607118

[19] Graus F Titulaer MJ Balu R Benseler S Bien CG Cellucci T. A clinical approach to diagnosis of autoimmune encephalitis. Lancet Neurol. (2016) 15:391–404. 10.1016/S1474-4422(15)00401-9 26906964 PMC5066574

[20] Baumgartner A Rauer S Mader I Meyer PT. Cerebral FDG-PET and MRI findings in autoimmune limbic encephalitis: correlation with autoantibody types. J Neurol. (2013) 260:2744–53. 10.1007/s00415-013-7048-2 23900756

[21] Heine J Prüss H Bartsch T Ploner CJ Paul F Finke C. Imaging of autoimmune encephalitis–Relevance for clinical practice and hippocampal function. Neuroscience. (2015) 309:68–83. 10.1016/j.neuroscience.2015.05.037 26012492

[22] Bacchi S Franke K Wewegama D Needham E Patel S Menon D. Magnetic resonance imaging and positron emission tomography in anti-NMDA receptor encephalitis: a systematic review. J Clin Neurosci. (2018) 52:54–9. 10.1016/j.jocn.2018.03.026 29605275

[23] Leypoldt F Buchert R Kleiter I Marienhagen J Gelderblom M Magnus T. Fluorodeoxyglucose positron emission tomography in anti-N-methyl-D-aspartate receptor encephalitis: distinct pattern of disease. J Neurol Neurosurg Psychiatry. (2012) 83:681–6. 10.1136/jnnp-2011-301969 22566598 PMC3740122

[24] Wenke NK Kreye J Andrzejak E van Casteren A Leubner J Murgueitio MS. N-methyl-D-aspartate receptor dysfunction by unmutated human antibodies against the NR1 subunit. Ann Neurol. (2019) 85:771–6. 10.1002/ana.25460 30843274 PMC6593665

[25] Ehrenreich H. Autoantibodies against the N-Methyl-d-Aspartate receptor subunit NR1: untangling apparent inconsistencies for clinical practice. Front Immunol. (2017) 8:181. 10.3389/fimmu.2017.00181 28298911 PMC5331041

[26] Bechter K. Encephalitis, mild encephalitis, neuroprogression, or encephalopathy-not merely a question of terminology. Front Psychiatry. (2019) 9:782. 10.3389/fpsyt.2018.00782 30787887 PMC6372546

[27] Castillo-Gomez E Kästner A Steiner J Schneider A Hettling B Poggi G. The brain as immunoprecipitator of serum autoantibodies against N-Methyl-D-aspartate receptor subunit NR1. Ann Neurol. (2016) 79:144–51. 10.1002/ana.24545 26505629

[28] Jarius S Paul F Aktas O Asgari N Dale RC de Seze J. MOG encephalomyelitis: international recommendations on diagnosis and antibody testing. J Neuroinflammation. (2018) 15:134. 10.1186/s12974-018-1144-2 29724224 PMC5932838

[29] Chang S O'Connor PM Slade BA Woo EJ. U.S. Postlicensure safety surveillance for adolescent and adult tetanus, diphtheria and acellular pertussis vaccines: 2005-2007. Vaccine. (2013) 31:1447–52. 10.1016/j.vaccine.2012.10.097 23142308

